# Spontaneous Fertility Following Conservative Surgical Management of Persistent Müllerian Duct Syndrome: A Case Report

**DOI:** 10.7759/cureus.108507

**Published:** 2026-05-08

**Authors:** Majid Karimi, Taha Karimi

**Affiliations:** 1 Urology, Zahedan University of Medical Sciences, Zahedan, IRN; 2 Biological Sciences, University of California San Diego, San Diego, USA

**Keywords:** conservative surgical management, disorder of sex development (dsd), indirect inguinal hernia, male infertility, persistent müllerian duct syndrome

## Abstract

Persistent Müllerian duct syndrome (PMDS) is a rare 46,XY disorder characterized by the presence of Müllerian structures in phenotypically normal males and is commonly associated with infertility. We report the case of a 28-year-old man with a seven-year history of primary infertility who underwent inguinal hernia repair. Intraoperatively, a uterus-like structure was identified, consistent with PMDS. A fertility-preserving approach was adopted, avoiding extensive dissection to prevent vas deferens injury. Bilateral orchiopexy and testicular biopsy were performed. Histopathology demonstrated active spermatogenesis without malignancy. At the seven-month follow-up, spontaneous conception was achieved without assisted reproductive techniques. This case highlights the importance of intraoperative diagnosis and individualized surgical management in optimizing fertility outcomes.

## Introduction

Persistent Müllerian duct syndrome (PMDS) is a rare 46,XY disorder of sex development, first described in 1939, with fewer than 300 reported cases [1-3]. It is inherited in an autosomal recessive pattern and most commonly results from mutations in the anti-Müllerian hormone (AMH) gene or its receptor (AMHR2) [2,4]. Normal Leydig cell function preserves male external genitalia and Wolffian duct derivatives, while failure of Müllerian duct regression results in the persistence of internal female reproductive structures [2,5].

PMDS typically presents as bilateral cryptorchidism, hernia uteri inguinalis, or transverse testicular ectopia and is often diagnosed incidentally during surgery [3,6,7]. Infertility is a common feature and is usually multifactorial, resulting from cryptorchidism, abnormal vas deferens anatomy, and potential iatrogenic injury during surgical intervention [6,8].

Spontaneous fertility in PMDS is rare, with most reported cases requiring assisted reproductive techniques [9,10]. We present a case of spontaneous conception following conservative surgical management in a patient with preoperative asthenoteratozoospermia. Given the rarity of spontaneous fertility and the ongoing considerations regarding optimal surgical management, reporting such cases is important to inform clinical decision-making.

## Case presentation

A 28-year-old man presented with a seven-year history of primary infertility. Family history suggested possible infertility in a paternal uncle. Physical examination revealed normal male external genitalia, a right inguinal bulge, and a left-sided hydrocele.

Semen analysis demonstrated asthenoteratozoospermia, with markedly reduced progressive motility (5%), severely abnormal morphology (<1% normal forms), and normal sperm concentration (45 million/mL). Semen volume (0.5 mL) and pH (6.5) were below reference ranges.

**Figure 1:**
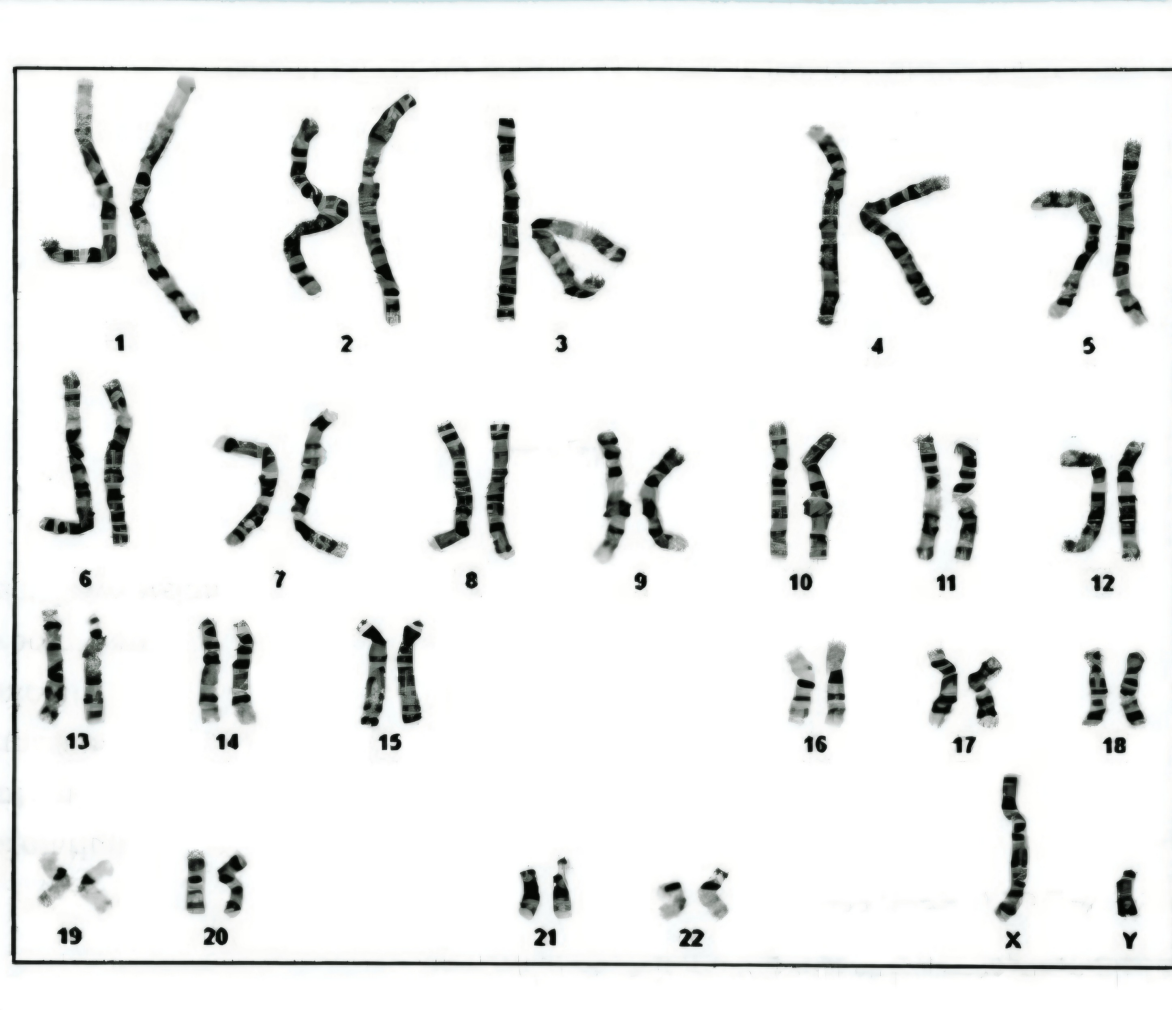
Karyotype analysis demonstrating a normal 46,XY chromosomal complement. Karyotype obtained from peripheral blood by GTG banding at 500–550 band resolution. Twenty metaphase spreads were analyzed; no chromosomal aberration was detected.

Chromosomal analysis confirmed a normal 46,XY karyotype (Figure 1).

Scrotal and inguinal ultrasonography demonstrated a Grade 2 left varicocele, mild left hydrocele, and a right inguinal hernia. Both testes were normal in size and echogenicity. No Müllerian structures or intra-abdominal testes were identified on preoperative imaging.

Based on these findings, the patient’s infertility was initially attributed to varicocele and inguinal hernia, and surgical repair was planned.

**Figure 2:**
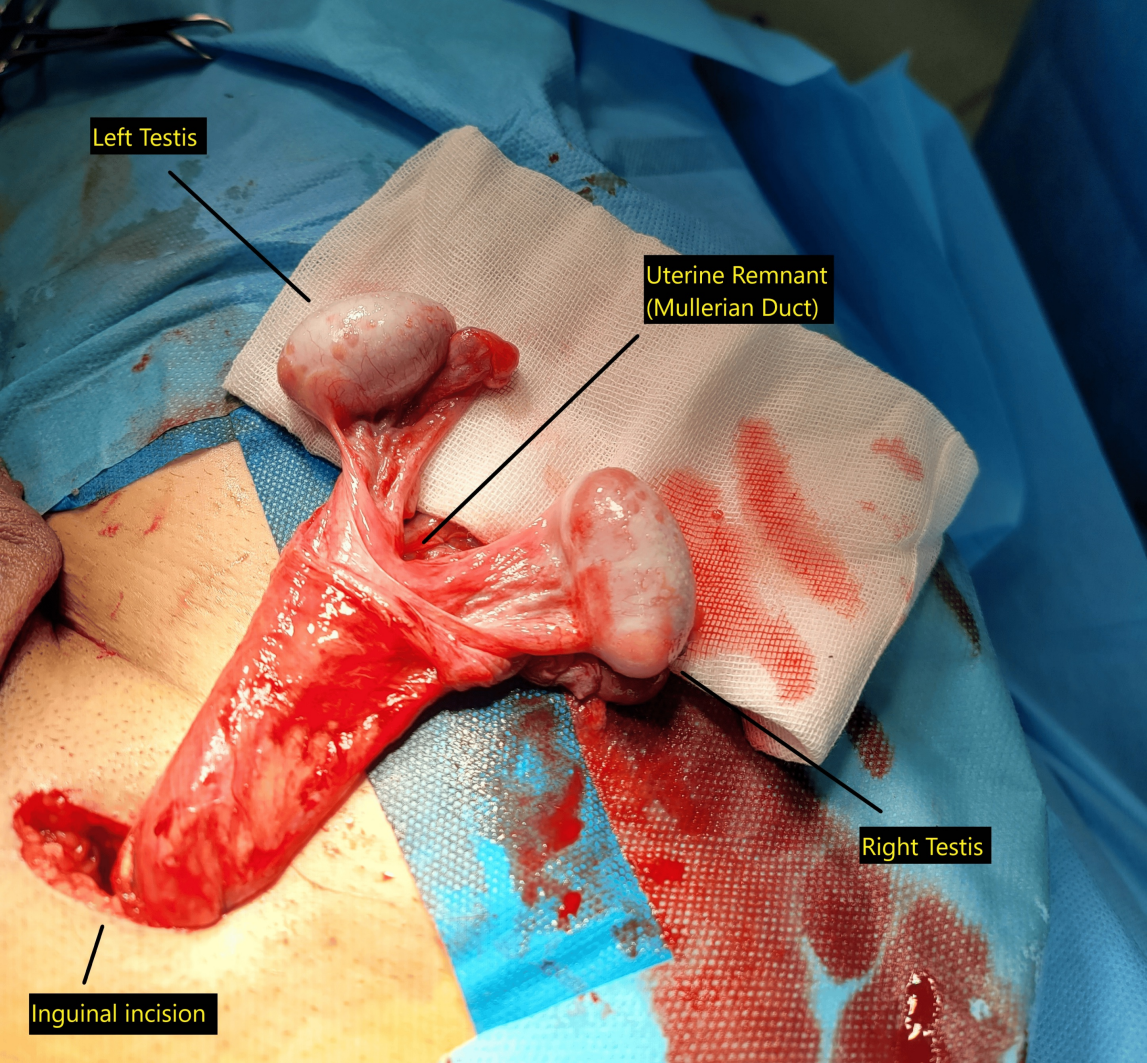
Intraoperative identification of persistent Müllerian duct structures during inguinal herniorrhaphy. A uterus-like Müllerian remnant with bilateral gonadal attachments identified during right inguinal herniorrhaphy. The left testis, right testis, and inguinal incision are labeled.

During right inguinal herniorrhaphy, a uterus-like Müllerian structure with bilateral gonadal attachments was identified, consistent with PMDS (Figure 2).

**Figure 3:**
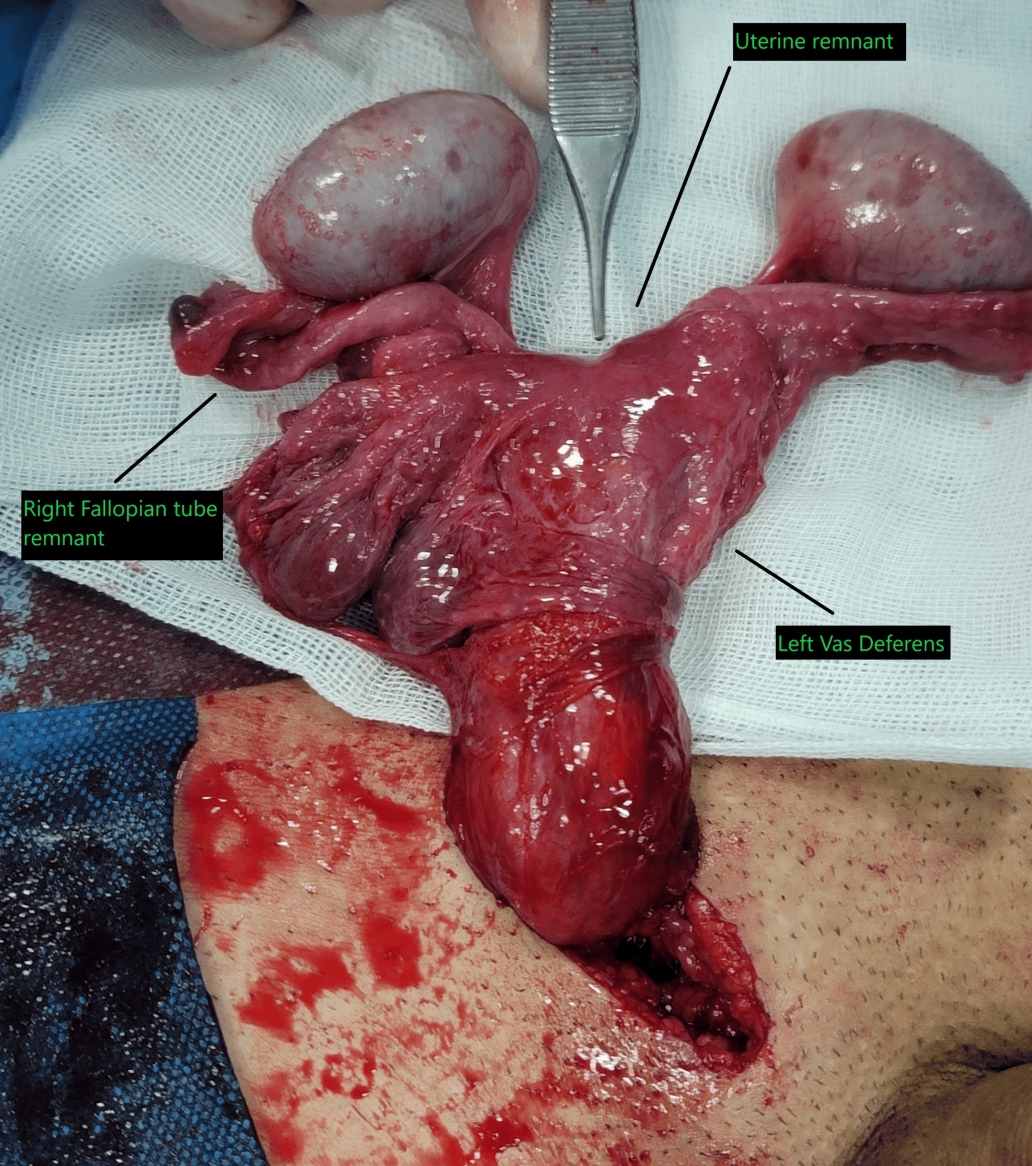
Close anatomical relationship between the vas deferens and Müllerian remnant. Detailed intraoperative view demonstrating the uterine remnant, right fallopian tube remnant, and left vas deferens. The intimate apposition of the vas deferens to the lateral wall of the Müllerian remnant is clearly visible. This anatomical relationship guided the decision to adopt a conservative, fertility-preserving approach rather than attempt resection of the Müllerian structures.

Given the patient’s desire for fertility and the close anatomical relationship between the vas deferens and the Müllerian remnant (Figure 3), a conservative, fertility-preserving approach was adopted. The Müllerian structures were preserved, and bilateral orchiopexy was performed.

**Figure 4:**
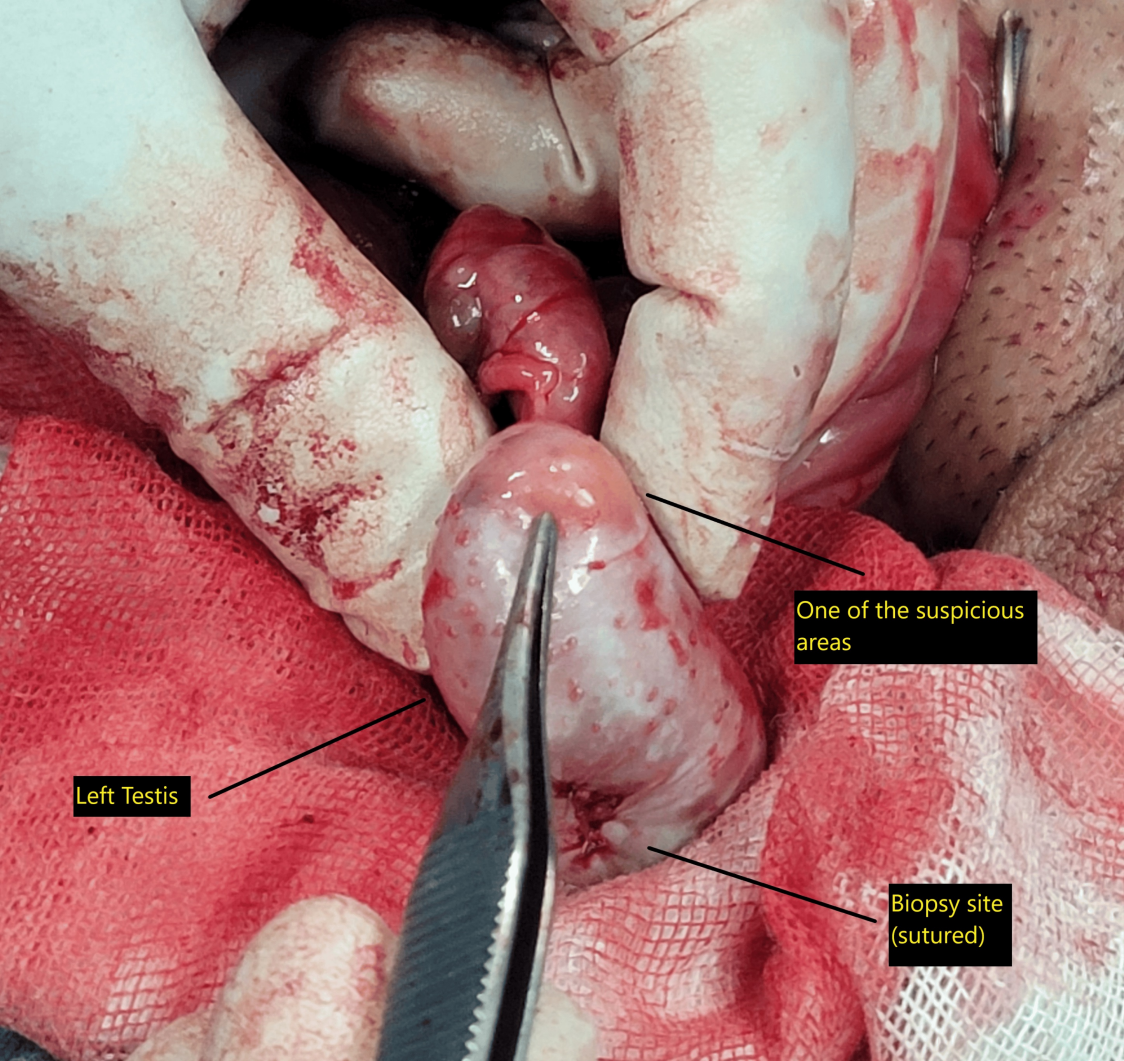
Suspicious areas on the left testicular surface. Intraoperative view of the left testis demonstrating two focal abnormalities raising concern for malignancy. One suspicious area is identified superiorly; a second, marked inferiorly, was biopsied and subsequently sutured.

Due to suspicious areas observed on the testicular surface, an incisional testicular biopsy was obtained (Figure 4). 

Histopathological examination demonstrated seminiferous tubules containing all stages of spermatogenesis with disorganized architecture, normal Leydig cells, and adjacent hyalinized fibrous tissue consistent with reparative change. No ovarian tissue or malignancy was identified.

The postoperative course was uneventful. At the seven-month follow-up, the patient reported spontaneous conception with his partner without the use of assisted reproductive techniques.

## Discussion

PMDS is difficult to diagnose preoperatively because the external genitalia are typically normal, and imaging studies often fail to identify Müllerian remnants [3,6,7]. In this case, ultrasonographic findings of varicocele, hydrocele, and inguinal hernia provided a sufficient explanation for the patient’s infertility, and the diagnosis of PMDS was made only intraoperatively. In our case, preoperative ultrasonography failed to identify Müllerian structures, consistent with prior reports highlighting the limited sensitivity of imaging in PMDS [3,6].

Preserved spermatogenesis on biopsy, despite preoperative asthenoteratozoospermia, suggests that infertility in this patient was likely multifactorial, potentially involving impaired sperm maturation or transport rather than primary testicular failure. Mechanical and thermal effects related to abnormal anatomy may have contributed to a suboptimal testicular environment [11]. 

The primary surgical dilemma in PMDS is whether to excise or preserve Müllerian structures. While excision may reduce the risk of malignancy, it carries a significant risk of vas deferens injury due to the close anatomical relationship between these structures [6,12]. A conservative approach, consisting of orchiopexy with preservation of Müllerian remnants, has been recommended in selected cases, particularly in patients desiring fertility [6,13-17].

In this patient, the decision to preserve Müllerian structures was guided by intraoperative findings demonstrating close adherence of the vas deferens. The subsequent spontaneous conception supports the effectiveness of a fertility-preserving strategy in appropriately selected cases.

Long-term follow-up is essential due to the potential risk of malignancy, and regular clinical and imaging surveillance is recommended [6,12,17,18].

A comprehensive hormonal evaluation was not performed preoperatively, as the patient was initially managed for inguinal hernia and infertility without suspicion of a disorder of sex development, which is consistent with standard clinical practice. Postoperative semen analysis was planned to further assess functional outcomes; however, repeat testing was not performed as the patient deferred evaluation despite follow-up. The occurrence of spontaneous conception during follow-up nevertheless provides indirect evidence of preserved reproductive potential.

## Conclusions

PMDS should be considered in adult males presenting with infertility and inguinal pathology, as preoperative diagnosis remains uncommon due to the limited sensitivity of imaging studies and the typically normal external phenotype. This case highlights the importance of intraoperative recognition of Müllerian structures and careful surgical decision-making. Management must be individualized, balancing the potential risk of malignancy against the risk of iatrogenic injury to the vas deferens. In patients with a strong desire for fertility and a close anatomical association between the vas deferens and Müllerian remnants, a conservative approach with orchiopexy and preservation of Müllerian structures may be appropriate. The successful spontaneous conception observed in this case underscores the potential for restoration of natural fertility even in patients with abnormal preoperative semen parameters. Increased awareness of PMDS among urologists and general surgeons is important for timely diagnosis and appropriate management. Long-term surveillance remains essential due to the ongoing risk of malignancy.
